# Association between Anti-Psychotic Drugs Use and Hip Fractures in Patients with Dementia: A Nationwide Population-Based Study

**DOI:** 10.3390/ijerph18158118

**Published:** 2021-07-31

**Authors:** Chia-Hung Tang, Yi-Chen Lai, Yi-Chen Chen, Shun-Min Chang, Yu-Han Chen, Jung-Yu Liao, Yi-Chi Wang, Chung-Han Ho, Ping-Jen Chen

**Affiliations:** 1 Department of Psychiatry, Tainan Hospital, Ministry of Health and Welfare, Tainan 700, Taiwan; yoyyotang@gmail.com; 2 Department of Emergency Medicine, An Nan Hospital, China Medical University, Tainan 709, Taiwan; johnnyliyijin@gmail.com; 3 Department of Medical Research, Chi Mei Medical Center, Tainan 710, Taiwan; laura751111986@hotmail.com; 4 Department of Orthopedics, Kaohsiung Municipal Ta-Tung Hospital, Kaohsiung Medical University, Kaohsiung 801, Taiwan; dtorth758@gmail.com; 5 Department of Family Medicine, Chi-Mei Medical Center, Tainan 710, Taiwan; smallquai@gmail.com; 6 Department of Public Health, Kaohsiung Medical University, Kaohsiung 807, Taiwan; jyliao@kmu.edu.tw; 7 Department of Family Medicine, Kaohsiung Municipal Hsiao-Kang Hospital, Kaohsiung Medical University, Kaohsiung 812, Taiwan; yichie1006@gmail.com; 8 Department of Information Management, Southern Taiwan University of Science and Technology, Tainan 710, Taiwan; 9 Division of Psychiatry, University College London, London W1T 7NF, UK; 10 Department of Family Medicine and Division of Geriatrics and Gerontology, Kaohsiung Medical University Hospital, Kaohsiung Medical University, Kaohsiung 807, Taiwan; 11 School of Medicine, Kaohsiung Medical University, Kaohsiung 807, Taiwan

**Keywords:** antipsychotic drugs, dementia, hip fracture, epidemiology, national health program

## Abstract

Background: People with dementia are a high-risk group for hip fractures. Although the increased risk of hip fractures associated with antipsychotic drugs (APD) is found in older populations, little is known about the risk for people with dementia living in Asia. We aimed to investigate the association between hip fractures and the characteristics of APD use in patients with dementia. Methods: A nested case-control analysis was conducted on a nationwide cohort in Taiwan. People with diagnoses of dementia during 2003–2012 were identified. Conditional logistic regression analysis was performed, and adjusted odds ratios (aORs) were calculated with a 95% confidence interval (CI) to estimate the risk of hip fractures. Results: APD use was associated with an increased risk of hip fractures in patients with dementia; current use or combined use of first and second generations of APDs had even higher risks. Regarding the duration of APD use, a U-shape curve of hip fracture risk was noted, and the risk peaked during 0–15 days and >215 days of exposure (aOR = 1.46, 95% CI 1.37–1.57; aOR = 1.47, 95% CI 1.37–1.58; respectively). Considering the doses of APDs, the hip fracture risk was significantly increased with all four levels of the cumulative doses and average daily doses and peaked in the group with the highest average daily dose. Conclusions: The findings suggest that caution must be taken when initiating APD use in patients with dementia, even in a small dose, and mixed types of APD prescriptions should be administered with care. Furthermore, frequent evaluation of the possibility of tapering or withdrawal of the medication is necessary, as the risk does not attenuate after long-term use.

## 1. Introduction

### 1.1. Background/Rationale

Hip fractures are not only devastating injuries that result in pain, decreased physical function, and dependence, but are also a major public health problems that can cause economic burden [1]. Hip fracture occurs more commonly in older people and causes increased morbidities, institutionalization, and mortality [2,3]. Compared with older people with normal cognitive function, people with dementia have a higher risk of hip fractures [4], recover less well after falls, and have approximately three times greater one-year mortality after hip fracture [5].

Anti-psychotic drugs (APDs) are widely used to treat several behavioral and psychological symptoms of dementia. However, they are reported to be associated with an increased risk of hip fracture [6,7,8] and mortality [9,10] among older people. It has been postulated that the side effects of APDs, such as sedation, orthostatic hypotension, and extrapyramidal symptoms (EPS), can all increase the risk of falls [11,12]. Furthermore, long-term use of APDs reduces bone mineral density (BMD) due to the prolactin-elevating effect [13,14], which may contribute to a higher probability of fracture resulting from a fall [15].

APDs are divided into first and second generations according to different pharmacological mechanisms. First-generation APDs (FGAs), also known as conventional or typical antipsychotics, mainly bind and inhibit dopaminergic D2 receptors and have significant potential to cause EPS and tardive dyskinesia. Second-generation APDs (SGAs), also known as atypical antipsychotics, bind to serotonergic receptors as well as dopaminergic receptors [16]. The existing literature has explored the association between APDs and hip fractures in different aspects. A recent meta-analysis conducted by Lee et al. revealed that the use of FGAs was associated with a higher risk of hip fractures (OR = 1.67; 95% CI 1.45–1.93), and the use of SGAs was associated with an attenuated but still significant risk of hip fractures (OR = 1.33; 95% CI 1.11–1.58) [6]. As for the exposure status of APDs, most research has revealed a higher risk of hip fracture in “current users” than in “past users” or “previous users” [7,17]. Research investigating the association between the duration of APD exposure and hip fractures has demonstrated an increased risk both shortly after the medication initiation and after a longer period of use [8,15,17], and some have reported an increased risk with a longer duration of use [7,18]. Despite the dose–response relationship between APD use and risk of hip fracture being an issue of interest, previous studies have not achieved conclusive results [17,19].

### 1.2. Objectives

The risk of hip fractures associated with APD use in the population with dementia has not been extensively explored, and the information about the influence of drug classes, duration, and dose of APDs on the risk in this patient group remains unclear. This study aims to investigate the association between hip fractures and exposure status, cumulative duration, average daily dose, and cumulative dose of APDs in patients with dementia.

## 2. Materialss and Methods

### 2.1. Data Source

The data for the present study were collected from the 2003 to 2012 National Health Insurance Research Database (NHIRD). For each National Health Insurance Program (NHIP) beneficiary, a unique encrypted identification number was used to retrieve sociodemographic background data from the NHIP registration records as well as ambulatory and inpatient services utilization in the NHI medical claim data files (including medical diagnosis, doses, duration of medications, and dates of visits). Our study protocol was approved by the institutional review board of Chi-Mei Medical Center in Taiwan (Approval No. 10410-E01), and informed consent was waived due to the anonymous nature of the NHIRD analysis.

### 2.2. Study Cohort

Patients who had at least three outpatient visits or one inpatient record with a diagnosis of dementia (International Classification of Diseases, Ninth Revision, Clinical Modification (ICD-9-CM) codes 290, 294, and 331) between 1 January, 2003 and 31 December, 2012 were identified from the NHIRD. Study cohort entry date was defined as the first dementia diagnosis date. We excluded patients aged less than 50 years old or who had a diagnosis of major fractures (ICD-9-CM codes 805, 806, 812, 813, 814, and 820 in at least one outpatient visit or inpatient record) before the cohort entry date. The patients with a history of previous flupentixol, sulpiride, and quetiapine usage before dementia diagnosis were also excluded, as these drugs have been frequently used off-label for anxiolysis, appetite, and sleep.

### 2.3. Cases and Controls

**Figure 1:**
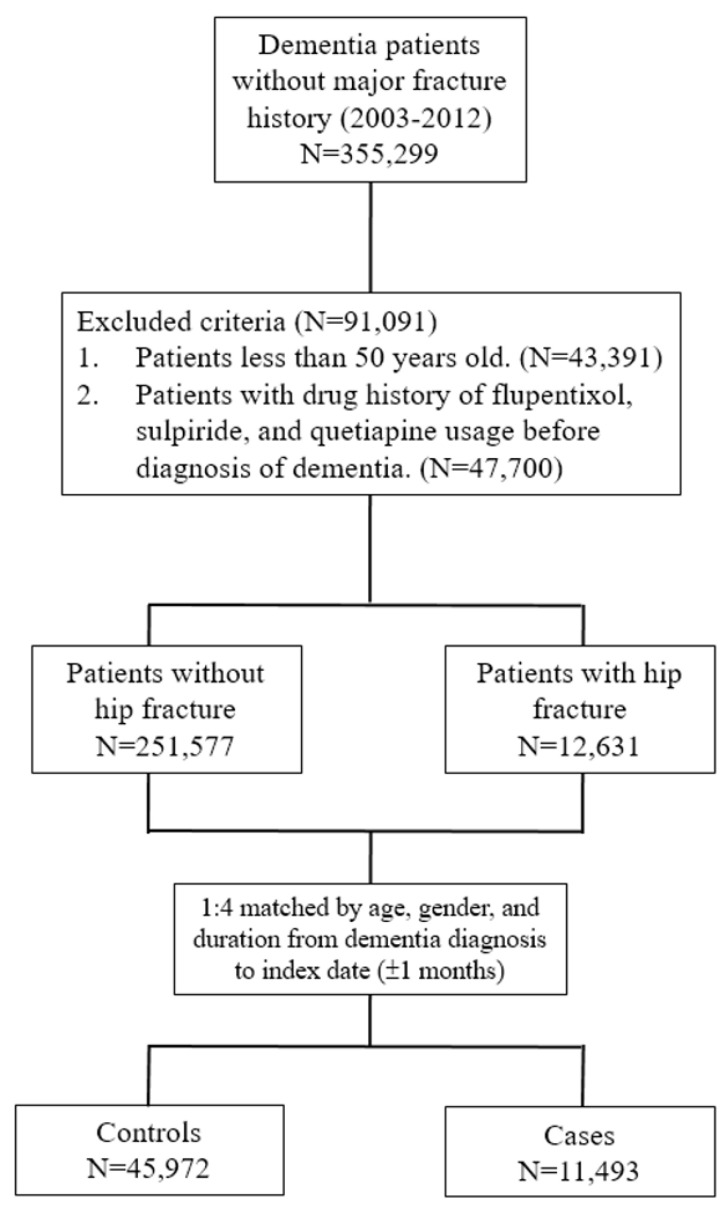
Schematic illustration of the study cohort and selection criteria of cases.

We defined the “cases” as patients who had a hip fracture diagnosis (ICD-9-CM code 820) in at least three outpatient visits or one inpatient record in the dementia cohort. The date of the first hip fracture diagnosis was defined as the outcome index date. For each case with hip fracture, four controls without hip fracture, matched by age, sex, and duration from the dementia diagnosis to hip fracture, were randomly selected from the same dementia cohort. The assigned index date of matched controls was the same as that of their matched case subjects (Figure 1).

### 2.4. Exposures

To investigate the association between APD and the risk of hip fracture, all prescriptions between the dementia diagnosis date and the outcome index date were retrieved. Both FGAs and SGAs were included (see Supplement Table S1).

We defined the categories of the status of APD exposure. “Nonusers” were defined as patients without prescriptions for APDs between dementia diagnosis date and outcome index date. “Current users” were defined as having prescriptions covering 0–30 days before the outcome index date. “Recent users” were defined as having prescriptions covering 31–180 days before the outcome index date. “Past users” were defined as having prescriptions covering >180 days before the outcome index date [7,17]. A sensitivity analysis of adding a 14-day grace period into each category of the status was also performed (see Supplement Table S2).

For analyzing the dose–response relationship between antipsychotic use and the risk of hip fracture, we defined cumulative duration, cumulative dose, and average daily dose. “Defined Daily Dose, DDD” is defined by the World Health Organization (WHO) as the assumed average maintenance dose per day for a drug used for its main indication in adults. The “cumulative duration” was calculated based on the prescription period between dementia diagnosis date and outcome index date (subdivided into 4 groups according to the quartile in a frequency distribution). The “cumulative dose” was calculated from all APD doses between dementia diagnosis date and outcome index date (subdivided into 4 groups according to the quartile in a frequency distribution). The “average daily dose” was the total cumulative dose divided by the total cumulative duration (≤0.5, 0.5–1, 1–2, >2).

### 2.5. Covariates

Comorbid medical/psychiatric diseases and concurrent medications were defined according to the codes in the period between 1 year before the dementia diagnosis date and the outcome index date (see Supplement Tables S1 and S3).

### 2.6. Statistical Analysis

Descriptive statistics of the hip fracture cases and matched controls were reported. Pearson’s chi-square test was used to examine the difference between the cases and controls. A *p*-value of less than 0.05 was considered statistically significant. To investigate the association between the use of APDs and the risk of hip fracture, conditional logistic regression analysis was done. Confounders, including comorbidities and concomitant medications, were adjusted, and adjusted odds ratios (aORs) were calculated with a 95% confidence interval (95% CI). The status of APD use, cumulative duration, cumulative dose, and average daily dose was also investigated. All analyses were performed using SAS version 9.4 (SAS Institute, Cary, NC, USA).

## 3. Results

We finally enrolled 11,493 cases with hip fracture and 45,972 controls in this study. The age and sex distributions of the cases and control subjects were well matched. The mean age of the subjects with dementia was 77.72 ± 7.94 years, and 55.70% were female. Regarding the medical comorbidity, cases were less likely to have hypertension, cerebrovascular disease, renal failure, hyperlipidemia, and arrhythmia. Regarding the psychiatric comorbidity, cases were more likely to have psychosis-related disorders, mood disorders, alcohol-related disorders, and sleep disorders, but less likely to have epilepsy. In addition, a higher proportion of cases than controls used anticholinergic agents, antidepressants, anxiolytics, hypnotics and sedatives, Z-drugs, mood stabilizers, oral glucocorticoids, hormone replacement therapy, and other osteoporosis medications (Table 1).

After adjusting for confounding factors, APDs were associated with a higher risk of hip fracture in patients with dementia (aOR = 1.38, 95% CI 1.32–1.45). The risk was slightly lower in the group of ≥80 age (50–65 age, aOR = 1.40, 95% CI 1.17–1.67; 65–80 age, aOR = 1.41, 95% CI 1.32–1.50; ≥80 age, aOR = 1.33, 95% CI 1.23–1.43) and in male group (female: aOR = 1.42, 95% CI 1.33–1.51; male: aOR = 1.33, 95% CI 1.24–1.43). Stratified by sex and age group, the highest risk was in 65–80 age female group (aOR = 1.45, 95% CI 1.34–1.58), and the lowest risk was in ≥80 age male group (aOR = 1.26, 95% CI 1.13–1.42) (Table 2).

**Figure 2:**
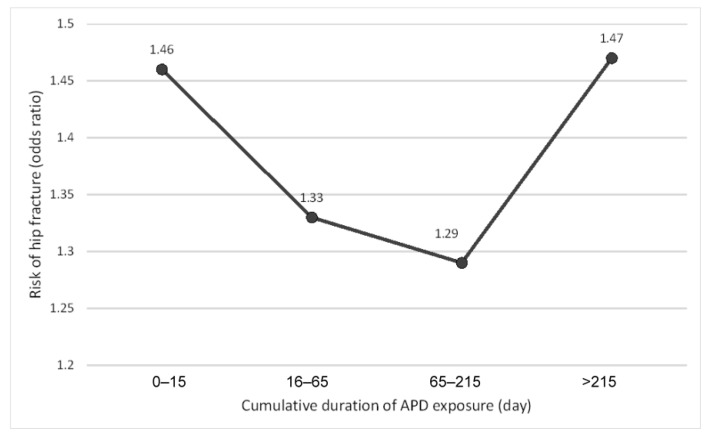
The risk of hip fracture in groups with different cumulative duration of antipsychotic drug (APD) exposure compared to APD nonusers.

Regarding the exposure status of APDs, “current users” had the highest risk of hip fractures (aOR = 2.36; 95% CI 2.23–2.50), with the second high risk in “recent users” (aOR = 1.23; 95% CI 1.14–1.33) (Table 3). The sensitivity analysis of adding a 14 day grace period into each category of the status showed a similar trend (see Supplement Table S2) Considering the types of APD, the group with combination use of FGA and SGA had the highest risk (aOR = 1.70; 95% CI 1.59–1.82), followed by the group that used SGA alone (aOR = 1.36; 95% CI 1.29–1.44) and FGA alone (aOR = 1.18; 95% CI 1.10–1.26). Regarding the cumulative duration of APD use, a U-shape curve of association was noted, and the risk of hip fracture peaked during 0–15 days and >215 days of exposure (aOR = 1.46, 1.33, 1.29, 1.47 in the four groups of cumulative duration) (Figure 2). Considering the cumulative doses of APDs, the hip fracture risk was significantly increased with all four levels of the dose, without a significant trend within groups (aOR = 1.33, 1.44, 1.37, 1.39 in the four groups of cumulative dose). A slightly increasing trend of the risk of hip fracture was noted in the stratification by average daily dose (aOR = 1.24, 1.34, 1.33, 1.44 in the four groups of average daily dose), and the risk significantly increased with the low amounts of exposure only in ≤0.5 DDD.

## 4. Discussion

To the best of our knowledge, this is the first nationwide population-based study to investigate the association between the risk of hip fracture and multiple detailed characteristics of APD exposure in patients with dementia. To facilitate the precision of clinical practice, the effects of dose response, including cumulative dose and average daily dose, on hip fracture risk were analyzed in this study; this was not done within the dementia patient group in previous studies. The results of this study are consistent with previous studies that found that APDs are associated with an increased risk of hip fractures [6,8,12,15,17,18,19,20].

### 4.1. APD and Risk of Hip Fractures

In line with the results in most previous research [7,12,17], we found a higher risk in current users compared to recent users and past users. The side effects that appear in the early stage of APD use, including postural hypotension, EPS, and sedative effect may be responsible for the increased risk of falls and fracture of current users [21,22]. However, the results need to be interpreted with caution because of the possible confounding by reverse causation, as fall events and hip fracture may increase the risk of psychosis, agitation, and delirium, leading to subsequent APD use. Furthermore, the potential indication bias should also be taken into account, because APD users may experience more complex behavioral and psychological symptoms of dementia (BPSD), which may contribute to the increased risk of falls and hip fracture. To minimize the influence of indication bias, we also performed a separate regression analysis, in which APD nonusers were excluded for different characteristics of antipsychotics exposure (Table 3).

Regarding the association between the risk of hip fracture and the cumulative duration of APD use, our results showed a remarkably increased risk developed in ≤15 days of exposure as well as another peak of risk when the cumulative duration exceeds 215 days. Similar results were also demonstrated in the existing literature. A case-control study conducted by Hugenholtz et al. showed that the risk of hip fracture increased immediately after therapy initiation and re-escalated with longer use [15]. Another case-control study also reported an increased risk during both the first eight months of APD use and when the duration of continuous use approached two years [17]. The first period of increased risk of fracture may reflect the influence of transient symptoms, such as orthostatic hypotension and parkinsonism, immediately after the initiation of APD use [21,22]. In addition, the hyperprolactinemia and subsequent reduced bone mineral density after several months of APD use [23,24,25] may be responsible for the second period of increased risk.

Our findings echo the American Psychological Association’s recommendation that APDs should only be used for the treatment of agitation and psychosis in patients with dementia when symptoms are severe or dangerous and the potential risk/benefit assessment favors their use. If clinically indicated, they should be initiated at a minimum effective dose, and the response to treatment should be regularly reviewed for early tapering and discontinuing of the medication [26].

A nested case-control study focusing on older patients with dementia living in nursing homes found no increased risk of hip fracture until the cumulative exposure exceeded six months [7]. However, the fact there were a small number of new users within that study may explain the results found. Our results also showed a slightly lower risk of hip fracture in the group of older age, which might be related to limited ambulatory function and high dependency for daily activity in older patients with dementia.

### 4.2. The Impact of Different Classes of APD on the Risk of Hip Fractures

The difference between FGAs and SGAs in increasing the risk of hip fracture has been investigated in various populations but has not seen conclusive results in previous studies. Jalbert et al. found a significant risk of hip fracture in the SGA group (OR = 1.27, 95% CI 1.05–1.54), and a higher but insignificant risk in the FGA group (OR = 1.44, 95% CI 0.84–2.47) in a nested case-control study [7]. Pratt et al. demonstrated a high risk of hip fracture in the first week post atypical APD exposure, whereas the risk associated with typical APDs in the same duration was insignificant (incidence rate ratio (IRR) = 2.17, 95% CI 1.54–3.06 vs. 1.04, 95% CI 0.40–2.70) in a self-controlled case-series study [27]. However, the risk associated with typical APDs exceeded atypical APDs after the second week of exposure in the study, which may reflect the different mechanisms of these two classes of drugs causing the hip fractures; the side effects of sedation and postural hypotension associated with SGAs occurs immediately after exposure, whereas the EPS associated with FGAs develops when treated for three to eight weeks [28]. Another case-control study on nursing home residents showed a significant risk of hip fracture in the SGA (OR = 1.37, 95% CI 1.11–1.69) and FGA group (OR = 1.35, 95% CI 1.06–1.71), with a mildly higher odds ratio in the SGA group [29]. Some population-based cohort studies involving older adults (aged >50 years) showed no significant difference between conventional and atypical APDs regarding the risk of fractures [18,30], whereas other studies [17,31] and a meta-analysis [32] demonstrated that FGAs were associated with a higher risk of hip fracture than SGAs.

Our results showed that the combined use of FGAs and SGAs was associated with the highest risk of hip fracture in people with dementia, followed by the group with SGA use alone, and finally by the group with FGA use alone. Although we should be aware of the indication bias, led by the patients who had the combined use of FGAs and SGAs and who may have been experiencing more severe BPSD and increased risk of fall and fracture, our results highlighted the importance of exercising caution with mixed classes of neuroleptic prescriptions in this vulnerable population. The postsynaptic blockade of dopamine D2 receptors is considered responsible for the extrapyramidal side effects and elevated serum prolactin levels [33,34]. Compared with FGAs, SGAs, which have a lower affinity for dopamine receptors [35], are generally associated with a lower risk of hip fracture. However, a meta-analysis revealed that only a few SGAs induce fewer extrapyramidal effects than FGAs [36]. The additional affinities for a variety of neurotransmitter receptors, including serotonin, histamine receptor H_1_, and adrenergic receptors, have contributed to multiple side effects, such as EPS, sedation, and postural hypotension [37,38]. In addition, with a few exceptions, all SGAs have the propensity to elevate prolactin levels above the upper limit of normal [34], and they may also affect bone density through other pathways [39,40].

### 4.3. The Dose–Response Relationship

Whether there is a dose–response relationship between the risk of hip fracture and APD use remains controversial according to the existing literature. A population-based study focusing on the schizophrenia population found a dose–response relationship between hip fracture and APD consumption [19]. In addition, Vestergaard et al. showed a higher dose of neuroleptics was associated with a higher risk of hip fracture in a nationwide case-control study [41]. The dose-related effects of APDs, such as parkinsonism, sedation, orthostatic hypotension, and hyperprolactinemia, may be responsible for these results [24,42]. A retrospective cohort study found no dose-dependent risk of falls/fractures in atypical APD use in older adults, but a significant loss of the sample and missing data in the study should be considered when interpreting this result [43].

Our results show that the risk of hip fracture increased significantly when the average daily exposure of APD was only ≤0.5 DDD/day. The risk slightly increased in the groups of higher average daily dose, peaking in the group with the highest daily dose (>2 DDD/day). The risk of hip fractures was significantly increased in all cumulative doses of APD, without an obvious trend. Therefore, in people with dementia, we cannot establish a dose–response relationship between the risk of hip fracture and APD use. Nevertheless, high dosages and chronic use of APDs should be prevented.

### 4.4. Strengths and Limitations

The strength of this study is the large sample size provided by Taiwan’s NHIRD. This is the first nationwide population-based study in patients with dementia to analyze the relationship between hip fracture and the characteristic details of APD use. Another strength is applying a matching method to increase the comparability between cases and controls. However, there are several limitations in our study. First, the NHIRD lacks comprehensive information on clinical variables, for example, bone marrow density, body mass index, physical activities, diet, smoking, laboratory data, and the severity of dementia. Instead, we collected proxy measures (for example, concomitant osteoporosis medications for bone marrow density) for the evaluation of these confounders. Second, the medication adherence information could not be obtained from the NHIRD. However, the nonadherence of medications may cause nondifferential misclassification and result in underestimation of the risk of hip fracture. Third, paralyzed or bedridden patients, wheelchair users, and patients with total dependence for activities of daily living were not adjusted due to the limitations of NHIRD information. Fourth, the severity of psychosis or agitation when the APD was prescribed was not obtainable from the NHIRD. This may result in potential indication bias. Last, the distinction between the risk of individual APD use and the different underlying mechanisms of APDs in the contribution to the hip fracture risks, such as short-term extrapyramidal symptoms/hypotension/sedation effects and long-term hyperprolactinemia/bone density effects, were not analyzed in this study.

## 5. Conclusions

We found that APD use was associated with a higher risk of hip fractures in patients with dementia, and the risk was significant even when APDs were used over a short duration, with a low average daily dose or cumulative dose. In addition, high dosage and mixed classes of APD prescriptions had an even higher increased risk. Non-pharmacological management should be attempted for symptom control in patients with dementia, and cautions must be taken when initiating APD use, even in a small dose. The combination of FGAs and SGAs should be administered with caution. Furthermore, frequent evaluation of the possibility of tapering or withdrawing APDs is necessary, as the risk of hip fracture does not attenuate after long-term use. Further large-scale, prospective and controlled studies with more detailed information of confounders are warranted. Exploring the association between different underlying mechanisms of APDs and their contributions to hip fracture risk in patients with dementia is required to facilitate the precision of clinical judgment of healthcare providers.

## Figures and Tables

**Table 1:** Demographics and characteristics of cases of hip fracture and their controls.

Characteristic	Hip Fracture N = 11,493	No Hip Fracture N = 45,972	*p*-Value
Age (Mean ± SD)	77.72 ± 7.94	77.72 ± 7.94	1.0000
50–65	839 (7.30)	3356 (7.30)	1.0000
65–80	6252 (54.40)	25,008 (54.40)	
≥80	4402 (38.30)	17,608 (38.30)	
Gender			
Female	6402 (55.70)	25,608 (55.70)	1.0000
Male	5091 (44.30)	20,364 (44.30)	
Comorbidity			
Anemia	581 (5.06)	2261 (4.92)	0.5445
Coronary heart disease	1839 (16.00)	7322 (15.93)	0.8464
Hypertension	5401 (46.99)	22,321 (48.55)	0.0028
Cerebrovascular disease	2859 (24.88)	13,545 (29.46)	<0.0001
COPD	1167 (10.15)	4918 (10.70)	0.0901
Diabetes	2535 (22.06)	10,182 (22.15)	0.8329
Malignant neoplasm	537 (4.67)	2231 (4.85)	0.4188
Peripheral vascular disease	52 (0.45)	243 (0.53)	0.3070
Parkinsonism	747 (6.50)	2773 (6.03)	0.0615
Rheumatoid arthritis	60 (0.52)	209 (0.45)	0.3435
Renal failure	433 (3.77)	1924 (4.19)	0.0435
Hyperlipidemia	1196 (10.41)	5159 (11.22)	0.0126
Menopause syndrome	99 (0.86)	480 (1.04)	0.0794
Obesity	5 (0.04)	16 (0.03)	0.6625
Arrhythmia	993 (8.64)	4336 (9.43)	0.0089
Thyrotoxicosis	46 (0.40)	204 (0.44)	0.5262
Psychiatry comorbidity			
Epilepsy	150 (1.31)	727 (1.58)	0.0307
Psychosis-related disorder	170 (1.48)	515 (1.12)	0.0015
Mood disorder	566 (4.92)	1961 (4.27)	0.0021
Alcohol-related disorder	61 (0.53)	149 (0.32)	0.0010
Substance use disorder	22 (0.19)	56 (0.12)	0.0699
Sleep disorder	1500 (13.05)	5286 (11.50)	0.0001
Concomitant drugs			
Anticholinergics	1629 (14.17)	4493 (9.77)	<0.0001
Antidepressants	3997 (34.78)	11,611 (25.26)	<0.0001
Anxiolytics	7659 (66.64)	24,066 (52.35)	<0.0001
Hypnotics and sedatives	3541 (30.81)	10,621 (23.10)	<0.0001
Z-drugs	3995 (34.76)	11,237 (24.44)	<0.0001
Mood stabilizers	1468 (12.77)	5146 (11.19)	<0.0001
Oral glucocorticoids	3902 (33.95)	13,415 (29.18)	<0.0001
HRT (HRT + SERM)	270 (2.35)	742 (1.61)	<0.0001
Other osteoporosis drugs	688 (5.99)	672 (1.46)	<0.0001

*p*-value was derived from Pearson’s chi-square test. SD = standard deviation; COPD = chronic obstructive pulmonary disease; HRT = hormone replacement therapy; SERM = selective estrogen receptor modulators.

**Table 2:** The risk of hip fracture in patients with and without antipsychotic drug exposure, stratified by age and sex.

Variable	Hip Fracture		No Hip Fracture		Crude OR (95% CI)	Adjusted OR ^a^ (95% CI)
	APD Non-Users	APD Users	APD Non-Users	APD Users		
Patients	4417 (38.43)	7076 (61.57)	23,620 (51.38)	22,352 (48.62)	1.73 (1.66–1.81)	1.38 (1.32–1.45)
Age groups						
50–65	327 (7.40)	512 (7.24)	1785 (7.56)	1571 (7.03)	1.83 (1.56–2.15)	1.40 (1.17–1.67)
65–80	2349 (53.18)	3903 (55.16)	12,713 (53.82)	12,295 (55.01)	1.77 (1.67–1.88)	1.41 (1.32–1.50)
≥80	1741 (39.42)	2661 (37.61)	9122 (38.62)	8486 (37.97)	1.67 (1.56–1.79)	1.33 (1.23–1.43)
Gender						
Female	2418 (37.77)	3984 (62.23)	13,154 (51.37)	12,454 (48.63)	1.79 (1.69–1.89)	1.42 (1.33–1.51)
50–65	142 (5.87)	222 (5.57)	766 (5.82)	690 (5.54)	1.79 (1.41–2.27)	1.35 (1.02–1.77)
65–80	1279 (52.89)	2208 (55.42)	7074 (53.78)	6874 (55.20)	1.84 (1.70–1.99)	1.45 (1.34–1.58)
≥80	997 (41.23)	1554 (39.01)	5314 (40.40)	4890 (39.26)	1.72 (1.57–1.88)	1.37 (1.24–1.51)
Male	1999 (39.27)	3092 (60.73)	10,466 (51.39)	9898 (48.61)	1.67 (1.57–1.78)	1.33 (1.24–1.43)
50–65	185 (9.25)	290 (9.38)	1019 (9.74)	881 (8.90)	1.87 (1.51–2.30)	1.41 (1.11–1.78)
65–80	1070 (53.53)	1695 (54.82)	5639 (53.88)	5421 (54.77)	1.70 (1.55–1.85)	1.36 (1.23–1.49)
≥80	744 (37.22)	1107 (35.80)	3808 (36.38)	3596 (36.33)	1.60 (1.44–1.77)	1.26 (1.13–1.42)

^a^ Adjusted by all confounding factors in Table 1. APD = antipsychotic drug; OR = odds ratio; CI = confidence interval.

**Table 3:** The risk of hip fracture according to different characteristics of antipsychotic drug exposure.

Characteristics of Antipsychotic Drug Exposure	Crude OR (95% CI)	Adjusted OR ^a^ (95% CI)	Adjusted OR ^b^ (95% CI)
Exposure status of APD			
Nonusers	Ref.	Ref.	-
0–30 (current users)	3.00 (2.85–3.16)	2.36 (2.23–2.50)	3.07 (2.84–3.32)
31–180 (recent users)	1.51 (1.40–1.63)	1.23 (1.14–1.33)	1.56 (1.41–1.73)
>180 (past users)	1.00 (0.95–1.06)	0.85 (0.80–0.90)	Ref.
Type of APD			
Nonusers	Ref.	Ref.	-
FGA alone	1.37 (1.28–1.46)	1.18 (1.10–1.26)	Ref.
SGA alone	1.61 (1.52–1.69)	1.36 (1.29–1.44)	1.17 (1.08–1.27)
Combination	2.37 (2.23–2.51)	1.70 (1.59–1.82)	1.55 (1.42–1.69)
Cumulative exposure dose (DDD)			
Nonusers	Ref.	Ref.	-
0–25	1.53 (1.44–1.64)	1.33 (1.24–1.42)	Ref.
26–225	1.76 (1.65–1.88)	1.44 (1.34–1.54)	1.10 (1.01–1.21)
226–1135	1.75 (1.64–1.86)	1.37 (1.28–1.47)	1.05 (0.96–1.15)
>1135	1.91 (1.79–2.03)	1.39 (1.30–1.49)	1.12 (1.02–1.22)
Cumulative exposure duration (day)			
Nonusers	Ref.	Ref.	-
0–15	1.67 (1.57–1.79)	1.46 (1.37–1.57)	Ref.
16–65	1.61 (1.51–1.71)	1.33 (1.24–1.42)	0.94 (0.85–1.03)
65–215	1.68 (1.57–1.79)	1.29 (1.21–1.38)	0.93 (0.85–1.02)
>215	2.01 (1.89–2.14)	1.47 (1.37–1.58)	1.07 (0.97–1.17)
Average daily dose (DDD/day)			
Nonusers	Ref.	Ref.	-
≤0.5	1.40 (1.29–1.52)	1.24 (1.14–1.35)	Ref.
0.5–1	1.59 (1.45–1.75)	1.34 (1.21–1.47)	1.05 (0.92–1.20)
1–2	1.64 (1.52–1.77)	1.33 (1.23–1.44)	1.10 (0.98–1.24)
>2	1.86 (1.77–1.95)	1.44 (1.37–1.52)	1.19 (1.08–1.31)

^a^ Adjusted for all confounding factors in Table 1. ^b^ Adjusted for all confounding factors in Table 1; the group of nonusers was excluded. APD = antipsychotic drug; OR = odds ratio; CI = confidence interval; Ref. = reference; DDD = defined daily dose; FGA = first-generation antipsychotic drugs; SGA = second-generation antipsychotic drugs.

## Data Availability

The data for this study was obtained from the NHIRD (http://nhird.nhri.org.tw/, accessed on 29 July 2021). The application for permission to access the data was sent from Chi-Mei Medical Center to National Health Research Institutes (NHRI), Taiwan and approved. But restrictions apply to the availability of these data, which were used under license for the Chi-Mei Medical Center and current study only, and so are not publicly available. Data are however can be checked for any researcher who may concern about its reliability upon reasonable request to the Department of Research or Institutional Review Board in Chi-Mei Medical Center, Taiwan (https://www.chimei.org.tw/main/cmh_department/top/54000_index.html, accessed on 29 July 2021). For further analyses of these data, researchers should apply for permission independently from the NHIRD, which has been transferred to the Health and Welfare Data Science Center (HWDC), Department of Statistics, Ministry of Health and Welfare, Taiwan (http://dep.mohw.gov.tw/DOS/np-2497-113.html, accessed on 29 July 2021).

## Supplementary Materials

The following are available online at https://www.mdpi.com/article/10.3390/ijerph18158118/s1, Table S1: Diagnosis and International Classification of Diseases, Ninth Revision, Clinical Modification (ICD-9-CM) codes, Table S2: Categories of drugs and drug names, Table S3: A sensitivity analysis of the risk of hip fracture according to exposure status of antipsychotic use.

## References

[1] Chang C.Y., Tang C.H., Chen K.C., Huang K.C., Huang K.C. (2016). The mortality and direct medical costs of osteoporotic fractures among postmenopausal women in Taiwan. Osteoporos. Int..

[2] Morri M., Ambrosi E., Chiari P., Orlandi Magli A., Gazineo D., D’Alessandro F., Forni C. (2019). One-year mortality after hip fracture surgery and prognostic factors: A prospective cohort study. Sci. Rep..

[3] Civinini R., Paoli T., Cianferotti L., Cartei A., Boccaccini A., Peris A., Brandi M.L., Rostagno C., Innocenti M. (2019). Functional outcomes and mortality in geriatric and fragility hip fractures-results of an integrated, multidisciplinary model experienced by the “Florence hip fracture unit”. Int. Orthop..

[4] Baker N.L., Cook M.N., Arrighi H.M., Bullock R. (2011). Hip fracture risk and subsequent mortality among Alzheimer’s disease patients in the United Kingdom, 1988–2007. Age Ageing.

[5] Shaw F.E. (2002). Falls in cognitive impairment and dementia. Clin. Geriatr. Med..

[6] Lee S.H., Hsu W.T., Lai C.C., Esmaily-Fard A., Tsai Y.W., Chiu C.C., Wang J., Chang S.S., Lee C.C. (2017). Use of antipsychotics increases the risk of fracture: A systematic review and meta-analysis. Osteoporos. Int..

[7] Jalbert J.J., Eaton C.B., Miller S.C., Lapane K.L. (2010). Antipsychotic use and the risk of hip fracture among older adults afflicted with dementia. J. Am. Med. Dir. Assoc..

[8] Koponen M., Taipale H., Lavikainen P., Tanskanen A., Tiihonen J., Tolppanen A.M., Ahonen R., Hartikainen S. (2017). Antipsychotic Use and the Risk of Hip Fracture Among Community-Dwelling Persons With Alzheimer’s Disease. J. Clin. Psychiatry.

[9] Schneider L.S., Dagerman K.S., Insel P. (2005). Risk of death with atypical antipsychotic drug treatment for dementia: Meta-analysis of randomized placebo-controlled trials. JAMA.

[10] Wang P.S., Schneeweiss S., Avorn J., Fischer M.A., Mogun H., Solomon D.H., Brookhart M.A. (2005). Risk of death in elderly users of conventional vs. atypical antipsychotic medications. N. Engl. J. Med..

[11] Neutel C.I., PSMaxwell C. (2002). Medication use and risk of falls. Pharmacoepidemiol. Drug Saf..

[12] Ray W.A., Griffin M.R., Schaffner W., Baugh D.K., Melton L.J. (1987). Psychotropic drug use and the risk of hip fracture. N. Engl. J. Med..

[13] Naidoo U., Goff D.C., Klibanski A. (2003). Hyperprolactinemia and bone mineral density: The potential impact of antipsychotic agents. Psychoneuroendocrinology.

[14] Chen C.Y., Lane H.Y., Lin C.H. (2016). Effects of Antipsychotics on Bone Mineral Density in Patients with Schizophrenia: Gender Differences. Clin. Psychopharmacol. Neurosci..

[15] Hugenholtz G.W., Heerdink E.R., van Staa T.P., Nolen W.A., Egberts A.C. (2005). Risk of hip/femur fractures in patients using antipsychotics. Bone.

[16] Richtand N.M., Welge J.A., Logue A.D., Keck P.E., Strakowski S.M., McNamara R.K. (2007). Dopamine and serotonin receptor binding and antipsychotic efficacy. Neuropsychopharmacology.

[17] Pouwels S., van Staa T.P., Egberts A.C., Leufkens H.G., Cooper C., de Vries F. (2009). Antipsychotic use and the risk of hip/femur fracture: A population-based case-control study. Osteoporos. Int..

[18] Mehta S., Chen H., Johnson M.L., Aparasu R.R. (2010). Risk of falls and fractures in older adults using antipsychotic agents: A propensity-matched retrospective cohort study. Drugs Aging.

[19] Sorensen H.J., Jensen S.O., Nielsen J. (2013). Schizophrenia, antipsychotics and risk of hip fracture: A population-based analysis. Eur. Neuropsychopharmacol..

[20] Dennis M., Shine L., John A., Marchant A., McGregor J., Lyons R.A., Brophy S. (2017). Risk of Adverse Outcomes for Older People with Dementia Prescribed Antipsychotic Medication: A Population Based e-Cohort Study. Neurol. Ther..

[21] Drici M.D., Priori S. (2007). Cardiovascular risks of atypical antipsychotic drug treatment. Pharmacoepidemiol. Drug Saf..

[22] Cunningham Owens D.G. (2014). A Guide to the Extrapyramidal Side-Effects of Antipsychotic Drugs.

[23] Meaney A.M., Smith S., Howes O.D., O’Brien M., Murray R.M., O’Keane V. (2004). Effects of long-term prolactin-raising antipsychotic medication on bone mineral density in patients with schizophrenia. Br. J. Psychiatry.

[24] Dogan Bulut S., Bulut S., Tuzer V., Ak M., Ak E., Kisa C., Aydemir C., Goka E. (2014). The Effects of Prolactin-Raising and Prolactin-Sparing Antipsychotics on Prolactin Levels and Bone Mineral Density in Schizophrenic Patients. Nöro Psikiyatr. Arşivi.

[25] Wieck A., Haddad P.M. (2003). Antipsychotic-induced hyperprolactinaemia in women: Pathophysiology, severity and consequences. Selective literature review. Br. J. Psychiatry.

[26] Reus V.I., Fochtmann L.J., Eyler A.E., Hilty D.M., Horvitz-Lennon M., Jibson M.D., Lopez O.L., Mahoney J., Pasic J., Tan Z.S. (2016). The American Psychiatric Association Practice Guideline on the Use of Antipsychotics to Treat Agitation or Psychosis in Patients With Dementia. Am. J. Psychiatry.

[27] Pratt N., Roughead E.E., Ramsay E., Salter A., Ryan P. (2011). Risk of hospitalization for hip fracture and pneumonia associated with antipsychotic prescribing in the elderly: A self-controlled case-series analysis in an Australian health care claims database. Drug Saf..

[28] Gao K., Kemp D.E., Ganocy S.J., Gajwani P., Xia G., Calabrese J.R. (2008). Antipsychotic-induced extrapyramidal side effects in bipolar disorder and schizophrenia: A systematic review. J. Clin. Psychopharmacol..

[29] Liperoti R., Onder G., Lapane K.L., Mor V., Friedman J.H., Bernabei R., Gambassi G. (2007). Conventional or atypical antipsychotics and the risk of femur fracture among elderly patients: Results of a case-control study. J. Clin. Psychiatry.

[30] Bakken M.S., Schjott J., Engeland A., Engesaeter L.B., Ruths S. (2016). Antipsychotic Drugs and Risk of Hip Fracture in People Aged 60 and Older in Norway. J. Am. Geriatr. Soc..

[31] Kolanowski A., FDWaller J.L., Ahern F. (2006). Outcomes of antipsychotic drug use in community-dwelling elders with dementia. Arch. Psychiatr. Nurs..

[32] Oderda L.H., Young J.R., Asche C.V., Pepper G.A. (2012). Psychotropic-related hip fractures: Meta-analysis of first-generation and second-generation antidepressant and antipsychotic drugs. Ann. Pharmacother..

[33] Stone J.M., Davis J.M., Leucht S., Pilowsky L.S. (2009). Cortical dopamine D2/D3 receptors are a common site of action for antipsychotic drugs—An original patient data meta-analysis of the SPECT and PET in vivo receptor imaging literature. Schizophr. Bull..

[34] Peuskens J., Pani L., Detraux J., De Hert M. (2014). The effects of novel and newly approved antipsychotics on serum prolactin levels: A comprehensive review. CNS Drugs.

[35] Divac N., Prostran M., Jakovcevski I., Cerovac N. (2014). Second-generation antipsychotics and extrapyramidal adverse effects. BioMed Res. Int..

[36] Leucht S., Corves C., Arbter D., Engel R.R., Li C., Davis J.M. (2009). Second-generation versus first-generation antipsychotic drugs for schizophrenia: A meta-analysis. Lancet.

[37] Nasrallah H.A. (2008). Atypical antipsychotic-induced metabolic side effects: Insights from receptor-binding profiles. Mol. Psychiatry.

[38] Burns M.J. (2001). The pharmacology and toxicology of atypical antipsychotic agents. J. Toxicol. Clin. Toxicol..

[39] Calarge C.A., Ivins S.D., Motyl K.J., Shibli-Rahhal A.A., Bliziotes M.M., Schlechte J.A. (2013). Possible mechanisms for the skeletal effects of antipsychotics in children and adolescents. Ther. Adv. Psychopharmacol..

[40] Collet C., Schiltz C., Geoffroy V., Maroteaux L., Launay J.M., de Vernejoul M.C. (2008). The serotonin 5-HT2B receptor controls bone mass via osteoblast recruitment and proliferation. FASEB J..

[41] Vestergaard P., Rejnmark L., Mosekilde L. (2006). Anxiolytics, sedatives, antidepressants, neuroleptics and the risk of fracture. Osteoporos. Int..

[42] Stroup T.S., Gray N. (2018). Management of common adverse effects of antipsychotic medications. World Psychiatry.

[43] Chatterjee S., Chen H., Johnson M.L., Aparasu R.R. (2012). Risk of falls and fractures in older adults using atypical antipsychotic agents: A propensity score-adjusted, retrospective cohort study. Am. J. Geriatr. Pharmacother..

