# Supplementary material for: Association between Anti-Psychotic Drugs Use and Hip Fractures in Patients with Dementia: A Nationwide Population-Based Study
Source: Int J Environ Res Public Health. 2021 Jul 31;18(15):8118. doi: 10.3390/ijerph18158118 (PMC8345939; doi:10.3390/ijerph18158118)
Supplement: Supplementary file 1 [file ijerph-18-08118-s001.zip › ijerph-1290495-supplementary.pdf]

International Journal of
*Environmental Research and Public Health*

MDPI

**Supplement table S1. Diagnosis and International Classification of Diseases, Ninth Revision, Clinical Modification (ICD-9-CM) codes**

| Diagnosis | ICD-9 codes |
|---|---|
| Hip fracture | 820 |
| Dementia | 290, 294, 331 |
| Anemia | 280 - 285 |
| Coronary heart disease | 410 - 414 |
| Hypertension | 401 - 405 |
| Cerebrovascular disease | 430 - 438 |
| Chronic obstructive pulmonary disease | 491, 492, 494, 496 |
| Diabetes mellitus | 250.x |
| Malignant neoplasm | 140 - 208 |
| Peripheral vascular disease | 441.x, 442.0, 785.4, V43.4 |
| Parkinsonism | 332.x |
| Rheumatoid arthritis | 714.0x |
| Renal failure | 584, 585, 586, 572.4 |
| Epilepsy | 345.x |
| Psychosis related disorder | 295.x, 297.x, 298.x |
| Mood disorder | 296.x, 300.4 |
| Alcohol related disorder | 291.x, 303.x, 357.5, 425.5, 535.3, 571.0, 571.1, 571.2, 571.3 |
| Substance use disorder | 292.x |
| Sleep disorder | 307.4x, 780.5x |

**Supplement table S2. Categoris of drugs and drug names**

| Categories of drugs | Drug names |
|---|---|
| First generation antipsychotic drugs (FGAs) | chlorpromazine, chlorprothixene, clopentixol, flupentixol, fluphenazine, haloperidol, levomepromazine, loxapine, moperone, penfluridol, perphenazine, pimozide, pipotiazine, prochlorperazine, thioridazine, thiothixene, trifluoperazine, zuclopenthixol |
| Second generation antipsychotic drugs (SGAs) | amisulpride, aripiprazole, clotiapine, clozapine, olanzapine, paliperidone, quetiapine, risperidone, sulpiride, ziprasidone, zotepine |
| Anticholinergics | benztropine, biperiden, orphenadrine, trihexyphenidyl |
| Antidepressants | agomelatine, amitriptyline, bupropion, citalopram, clomipramine, dosulepin, doxepin, duloxetine, escitalopram, fluoxetine, fluvoxamine, imipramine, maprotiline, mianserin, milnacipran, mirtazapine, moclobemide, oxitriptan, paroxetine, sertraline, trazodone, venlafaxine, viloxazine |
| Anxiolytics | alprazolam, bromazepam, buspirone, chlordiazepoxide, clobazam, clonazepam, diazepam, fludiazepam, hydroxyzine, lorazepam, medazepam, mephenoxalone, meprobamate, nordazepam, oxazepam, oxazolam, potassium clorazepate, prazepam |
| Hypnotics and sedatives | amobarbital, bromisoval, brotizolam, dexmedetomidine, estazolam, flunitrazepam, flurazepam, lormetazepam, midazolam, nimetazepam, nitrazepam, pentobarbital, proxibarbal, secobarbital, temazepam, triazolam |
| Z drugs | eszopiclone, zaleplon, zolpidem, zopiclone |
| Mood stabilizers | acetazolamide, carbamazepine, gabapentin, lamotrigine, levetiracetam, lithium, oxcarbazepine, pregabalin, topiramate, valproic acid, zonisamide |
| Oral glucocorticoids | betamethasone, cortisone, dexamethasone, hydrocortisone, methylprednisolone, paramethasone, prednisolone, triamcinolone |
| Hormone replacement therapy (HRT) | estradiol, estriol, conjugated estrogens, estrogens, diethylstilbestrol , estradiol combinations, methyltestosterone and estrogen, testosterone and estrogen, Androgen progestogen and estrogen in combination, norethisterone and estrogen , hydroxyprogesterone and estrogen, ethisterone and estrogen , progesterone and estrogen , norgestrel and estrogen, medroxyprogesterone and estrogen , cyproterone and estrogen |
| Selective estrogen receptor modulator (SERM) | raloxifene, bazedoxifene |
| Other osteoporosis drugs | etidronate, clodronate, pamidronate, alendronate, ibandronic acid, risedronate, zoledronic acid, alendronic acid and colecalciferol, calcitonin, teriparatide, denosumab |

**Supplement table 3.** A sensitivity analysis of the risk of hip fracture according to exposure status of antipsychotic use

| Characteristics of antipsychotics exposure | Crude OR (95% C.I.) | Adjusted ORa (95% C.I.) | Adjusted ORb (95% C.I.) |
|---|---|---|---|
| **Exposure status of antipsychotic use** | | | |
| **Nonusers** | Ref. | Ref. | - |
| **0-45 (current users with grace period)** | 2.87 (2.73-3.02) | 2.26 (2.13-2.39) | 2.97 (2.75-3.21) |
| **46-195 (recent users with grace period)** | 1.49 (1.38-1.62) | 1.21 (1.12-1.32) | 1.52 (1.37-1.69) |
| **>195 (past users with grace period)** | 0.99 (0.94-1.05) | 0.84 (0.79-0.89) | Ref. |
